# Acute Appendicitis in the Elderly: A Nationwide Retrospective Analysis

**DOI:** 10.3390/jcm13072139

**Published:** 2024-04-08

**Authors:** Malkiely Gal, Paran Maya, Kobo Ofer, Khan Mansoor, Abbou Benyamine, Kessel Boris

**Affiliations:** 1 Division of Surgery, Hillel Yaffe Medical Center, Hadera 38100, Israel; 2 Department of Pediatric and Adolescent Surgery, Schneider Children’s Medical Center, Petah Tikva 4920235, Israel; paran.maya@gmail.com; 3 Division of Cardiology, Hillel Yaffe Medical Center, Hadera 38100, Israel; 4 Department of Major Trauma, Hull University Teaching Hospitals, Hull HU3 2JZ, UK; 5 Hospital Administration Hillel Yaffe Medical Center, Hadera 38100, Israel; benyaminea@hymc.gov.il; 6 Rappaport Faculty of Medicine, Technion-Israel Institute of Technology, Haifa 31096, Israel

**Keywords:** acute appendicitis, elderly, comorbidities, clinical outcomes, age-related complications

## Abstract

**Background:** Acute appendicitis (AA) in older individuals remains understudied. We aimed to assess AA characteristics in patients older than 60 years and evaluate the impact of comorbidities. **Methods:** This retrospective study analyzed data from the American National Inpatient Sample between 2016 and 2019 to compare AA characteristics in patients younger and older than 60 years. **Results:** Of the 538,400 patients included, 27.5% were older than 60 years. Younger patients had a higher appendectomy rate (*p* < 0.01), while the complicated appendicitis rate was higher in older patients. Superficial wound infection, systemic infection, and mortality rates were higher in older patients (*p* < 0.01). Risk factors for superficial wound infection in patients younger than 60 years included cerebrovascular disease, chronic kidney disease, hypertension, heart failure, and obesity, whereas only heart failure was a risk factor in older patients. Risk factors for systemic infection in young patients included hypertension, heart failure, obesity, and diabetes mellitus, while in older patients they included hypertension, heart failure, and obesity. Complicated appendicitis was not a risk factor for infections in either group. **Conclusions:** This study highlights a higher incidence of AA in older individuals than previously reported, with comorbidities posing differing risks for infections between age groups.

## 1. Introduction

Acute appendicitis (AA) represents a prevalent surgical condition, with an overall lifetime risk of 7–8% [1]. Historically, AA has predominantly been studied in young patients, with research primarily focusing on this population due to the perceptions of AA as a disease primarily affecting the young population. For example, in a study evaluating the significance of preoperative in-hospital delay on appendiceal perforation, the maximal reported patient age did not exceed 47 years [2]. Similarly, this age limitation was reported in other previous studies, which focused on the efficacy of non-operative management of AA [3,4]. Conversely, only a few studies evaluated AA and the perforation risk in non-young patients, such as the study by Omari et al. which included patients older than age 60 [5]. According to a review of the literature, the real incidence of acute appendicitis in non-young patients remains under-investigated. This gap in research may stem from the lack of a standardized, clear definition of the term “elderly” patients within the medical and surgical literature, resulting in a somewhat arbitrary age range for inclusion in studies investigating AA, ranging from 50 to 75 years [6,7]. As a result of this existing bias, the true clinical outcomes, complications, and mortality associated with AA in the elderly population remain unclear, posing challenges for decision-making processes in AA management. This becomes particularly pertinent in the contemporary landscape of non-operative management approaches to AA and the consideration of safety in delays of surgery. Within the spectrum of AA, distinct patient subgroups exist, each presenting with marked clinical, therapeutic, and prognostic differences. For example, the pediatric population, which comprises a distinct and separate subgroup of patients with AA, has been extensively investigated in the last decade, leading to significant advancements in understanding etiopathogenesis, diagnosis, and treatment [8,9]. Currently, the adult population, especially the elderly subset, remains comparatively understudied, with a lack of sufficient data. Therefore, a targeted effort to initiate studies focusing on the elderly population is needed. In light of these considerations, this study utilized a large American database to address these gaps in medical research and knowledge. The primary objective of the study was to evaluate the incidence of various forms of AA in patients, stratified by age, particularly comparing those younger and older than 60 years. The secondary aim of this study was to compare rates of surgical complication and mortality between these two age groups and try to identify specific risk factors for morbidity in the different populations.

## 2. Patients and Methods

### 2.1. Study Design

This retrospective study utilized data from the National Inpatient Sample (NIS) to investigate acute appendicitis in adults aged 18 years and older in the United States. The NIS is the largest publicly accessible all-payer inpatient care database, covering over 7 million hospital stays. Data collection was available between 1 January 2016 and 31 December 2019. 

### 2.2. Patient Selection

Patients with a diagnosis of AA were identified using the International Classification of Disease, Tenth Revision (ICD-10) codes. Inclusion criteria included all patients aged 18 years and older with a diagnosis of AA. Patients with an ICD-10 code of K35.890 were classified as uncomplicated appendicitis. All other patients were categorized as generalized peritonitis, local peritonitis, and gangrenous appendicitis according to the relevant ICD-10 codes. Patients categorized as other and unspecified appendicitis (ICD-10 codes of K36 and K37) were excluded. Patients were stratified into two primary groups: Group A (aged 18–60 years) and Group B (aged over 60 years). Subsequently, Group B was further divided into two subgroups: Group B1 (aged 60–70 years) and Group B2 (aged over 70 years).

### 2.3. Data Collection and Analysis

Data collected included patient demographics (age, gender), diagnosis (including subtypes of AA according to the ICD-10 codes), presence of comorbidities and chronic diseases, length of hospital stay, complications, and mortality. When analyzing results for clinical outcomes, (including length of hospital stay, mortality, superficial wound infection, and systemic infection), only patients who underwent an appendectomy were included. A comparison between Group A and Group B was performed with regard to demographics, existing comorbidities, and clinical outcomes. Following the comparison between the two main study groups, a sub-analysis was performed for Group B, comparing patients 60–70 years old and patients older than 70 years. In addition, a univariate analysis of comorbidities as risk factors for superficial and systemic infection was performed separately for Groups A and B. Following the univariate analysis, a multivariate analysis for infection risk factors was performed for each group separately.

### 2.4. Statistical Analysis

A comparison between Groups A and B and a sub-analysis comparison between Groups B1 and B2 were performed for baseline and studied variables. A Shapiro–Wilk test was performed for each continuous variable to determine whether it was normally distributed. Accordingly, continuous variables were compared using an independent sample *t*-test or a Mann–Whitney test, as required. A Pearson X2 test or Fisher exact test was performed for categorical variables, as needed. Following the univariate analysis, a multivariate logistic regression model for superficial wound infection and systemic infection was constructed to determine risk factors, in each study group separately. Statistical significance was considered as a two-tailed *p*-value of 0.05 or less. All analyses were performed using IBM SPSS Statistics version 29 software. 

## 3. Results

### 3.1. Demographics, Chronic Health Conditions, and Disease Characteristics

During the study period, 538,400 patients were registered in the NIS database. Overall, 279,195 patients (51.9%) were males, and 535,850 (99.5%) patients had complicated AA. Group A included 390,050 patients 18–60 years old, and Group B included 148,350 patients older than 60 years. Overall, 445,975 (82.8%) patients underwent appendectomy, and 92,425 (17.2%) were treated conservatively. In Group A, 334,270 (85.7%) patients underwent appendectomy, and 332,500 (99.4%) of them had complicated appendicitis. In Group B, 111,705 (75.3%) patients underwent appendectomy, and 111.325 (99.6%) of them had complicated appendicitis. The rate of appendectomy versus conservative treatment was higher in Group B compared to Group A (*p*-value < 0.01). Overall, percutaneous drainage was performed in 7150 (1.3%) patients who were not operated on. In comparison with younger patients (Group A), older patients (Group B) were less likely to be male, less likely to undergo an appendectomy, and more likely to have complicated appendicitis. Additionally, patients in Group B were more likely to suffer from chronic health conditions, including dementia, cerebrovascular disease, chronic kidney disease, hypertension, heart failure, obesity, and diabetes mellitus.

The results for demographics, chronic health conditions, and rates of complicated AA are presented in Table 1.

### 3.2. Clinical Outcomes

Of the overall patients who underwent appendectomy, 850 patients (0.2%) had a superficial wound infection, and 3370 patients (0.8%) had a systemic infection. The overall mortality rate in the study was 0.2%. Superficial wound infection and mortality were more common in Group B (*p* < 0.01). Systemic infection was more common in Group B in patients with complicated appendicitis (*p* < 0.01), but not in patients with uncomplicated appendicitis. The average length of hospital stay was longer in Group B (*p* < 0.01). The results for clinical outcomes are presented in Table 2. 

### 3.3. Sub-Analysis of Clinical Outcome in Group B

Among all patients in Group B, 77,985 (52.6%) patients were 60–70 years old (Group B1), and 70,365 (47.4%) patients were older than 70 years (Groups B2). In Group B1, 61,130 (78.6%) patients underwent appendectomy, of them 60,965 (99.7%) had complicated appendicitis. In Group B2, 50,575 (71.9%) patients underwent appendectomy, of them 50,360 (99.6%) had complicated appendicitis. The results of the sub-analysis of clinical outcome in Group B are presented in Table 3. 

### 3.4. Risk Factors for Superficial Wound Infection and Systemic Infection

The results for the association between different chronic health conditions and superficial wound infection as well as systemic infection, for both study groups, are presented in Table 4. In Group A, cerebrovascular disease, chronic kidney disease, hypertension, heart failure, obesity, and diabetes mellitus were all more common in patients with superficial wound infection. All of the abovementioned chronic conditions were also more common in patients with systemic infection in Group A, other than cerebrovascular disease. In Group B, rates of heart failure were higher in patients with superficial wound infection, and rates of hypertension, heart failure, and obesity were higher in patients with systemic infection in Group B. The results of the multivariate analysis for risk factors for superficial wound infection and systemic infection in both study groups are presented in Table 4. 

The results of the multivariate analysis for risk factors for superficial wound infection and systemic infection are presented in Table 5. Cerebrovascular disease, chronic kidney disease, hypertension, heart failure, and obesity were all identified as individual risk factors for superficial wound infection in patients younger than 60. Hypertension, obesity, and diabetes mellitus were recognized as individual risk factors for systemic infection in patients younger than 60. Heart failure was identified as an individual risk factor for superficial wound infection in patients older than 60. Hypertension, heart failure, and obesity were identified as individual risk factors for systemic infection in patients older than 60.

## 4. Discussion

In this study, we set out to explore the occurrence and clinical outcomes of AA in older adults, contrasting them with outcomes in younger patients. Our investigation encompassed a diverse range of ages, extending from 18 to 90 years, allowing for a thorough examination of AA across different age groups. The primary aim was to assess the incidence of AA, analyze surgical complications and mortality rates across age groups, and assess the role of existing comorbidities as risk factors for complications in patients in different age groups. 

The true incidence of AA in older individuals remains unknown. The variability of reported rates of AA in the elderly most probably results from a lack of a clear and standardized definition of the term “elderly” across studies. The relevant existing medical literature shows varying age cutoffs, ranging from 50 to 75 years, with the highest reported incidence rate of AA in elderly patients being 15% [7,10,11]. In contrast, our study, which included a substantial sample size, revealed a significantly higher incidence of AA in older patients (27%) compared with all previously published studies. This notable difference likely mirrors the global trend of population aging and underscores the importance of recognizing AA as a potential diagnosis across all age groups. Our study also conducted a detailed comparison between patients within Group B, specifically those aged 60–70 years and those older than 70 years. Our results revealed a higher incidence of superficial wound infections among patients older than 70 years compared to their counterparts aged 60–70 years. However, surprisingly, no such significant difference was observed in the incidence of systemic infections between these age groups. In addition, patients older than 70 years exhibited a higher mortality rate compared to patients aged 60–70 years. Moreover, the mean length of hospital stay following complicated appendicitis was notably longer in older patients. These findings, together with our findings regarding the comparison between patients younger and older than 60 years (Group A vs. Group B), suggest an age-dependent effect on the clinical outcome of patients with appendicitis. 

The increased incidence of AA in the non-young group that was found in the current study has a significant impact on the characteristics of this disease. The possible “changing face” of AA may be reflected in the future in different diagnostic and therapeutic approaches that should be developed after future high-quality prospective studies. For comparison, there are many studies assessing AA in the counterpart pediatric population. In this group of patients, a clear paradigm shift in the treatment of AA has developed in recent years after non-operative management has been established. Another good example is the use of artificial intelligence in the diagnosis of AA in children compared with single studies in adults [12]. 

Of particular interest is the remarkably high rate of complicated AA observed in our study compared to previous reports, which documented significantly lower rates. For example, in a study on 4962 patients with appendicitis, Solis Pazmino reported only a 38% prevalence of complicated appendicitis [13]. Similar results were also reported by other authors [12,14]. Similarly, specifically in the elderly group, Weinandt and coauthors reported a rate of 64% for complicated AA. This difference in rates of complicated AA, regardless of patients’ age, warrants further investigation. Possible explanations include delayed hospital presentation due to significant travel distances; prolonged processes of preoperative diagnosis, imaging, and treatment [8,15]; and operating theater delays [16], especially in tertiary centers. In addition, we speculate that surgeons tend to “aggravate” the description of operative findings, which may explain the high rates of reported complicated appendicitis. On the other hand, the large number of participating patients in this study possibly allows us to exclude this explanation. 

The impact of chronic health conditions on the development of surgical site infection (SSI), particularly following appendectomy, remains poorly understood. Previous studies focusing on clean surgeries like spinal procedures [17] identified obesity as an independent risk factor for both deep and superficial SSI; additionally, a study on 343 patients with SSI found diabetes mellitus and obesity as independent predictors of SSI [18]. Similar findings were reported in a meta-analysis of multiple surgical procedure types [19]. However, the role of comorbidities in clean-contaminated surgeries, such as appendectomy, is less clear, and this field currently lacks high-quality research. Our study contributes to this knowledge gap by identifying specific comorbidities associated with increased SSI risk in both younger and older patients. Notably, while younger patients exhibited a broader range of comorbidities as risk factors for SSI, older patients demonstrated fewer individual risk factors. With increasing age, comorbidities become more frequent among patients, but interestingly, not all comorbidities listed by the American Society of Anesthesiologists (ASA) are associated with an increased rate of complications. For example, in his study, Renteria demonstrated that appendectomy in elderly veteran patients has a low complication rate, similar to that in younger patients [20]. In another study on elderly patients, the authors found that independent factors for postoperative complications after appendectomy included anemia, positive history of cardiac disease, chronic renal insufficiency, and open appendectomy. However, after adjustment, the only independent predictor of postoperative morbidity was preoperative creatinine level [21]. Previous studies investigating the impact of comorbidities on surgical patients’ clinical outcomes and infections reported several key observations. Boehme et al. highlighted how patient comorbidities significantly increased postoperative resource utilization following both laparoscopic and open cholecystectomy procedures. This study reported that heart failure, ischemic heart disease, cirrhosis, diabetes mellitus, and hypertension were all significantly associated with postoperative emergency department visits and readmissions following open and laparoscopic cholecystectomy [22]. Similarly, Park et al. reported on the clinical outcomes of octogenarians undergoing laparoscopic cholecystectomy for acute cholecystitis, emphasizing the influence of preoperative disease severity and comorbidities on patient outcomes. Their results showed pulmonary comorbidities were an independent risk factor for complications post cholecystectomy [23]. Cox et al. explored the cost implications of preventable comorbidities on wound complications in open ventral hernia repair, emphasizing the economic burden associated with comorbidity-related complications. The study found that patients with diabetes mellitus and/or obesity were more likely to suffer wound-related complications after open ventral hernia repair [24]. These studies collectively underscore the importance of considering comorbidities in surgical patients and highlight the potential impact on postoperative outcomes and resource utilization. Our study further contributes to this body of evidence by examining the specific influence of comorbidities on surgical site infections in patients with acute appendicitis, providing valuable insights into risk stratification and management strategies in this patient population. However, in our study, not all significant comorbidities were found to be individual risk factors for SSI in patients older than 60 years. Interestingly, risk factors for superficial wound infection in Group A included cerebrovascular disease, chronic kidney disease, hypertension, heart failure, and obesity, whereas only heart failure was a risk factor in Group B. With regard to systemic infection, hypertension, heart failure, obesity, and diabetes mellitus were found to be individual risk factors in patients younger than 60, while only hypertension, heart failure, and obesity were found to be individual risk factors in older patients. We may only assume that younger patients had more severe and less controlled comorbidities. Another possible explanation for younger patients exhibiting a broader range of comorbidities as risk factors for SSI, compared to older patients who demonstrated fewer individual risk factors, is a modified age-related immuno-humoral response [25]. Further research is needed to elucidate the underlying mechanisms driving these differences. By elucidating the incidence, outcomes, and risk factors associated with AA in older individuals, this study endeavors to contribute to a deeper understanding of the disease in a demographic traditionally underrepresented in the literature. The interesting results of our work warrant prospective studies to build upon our findings and provide deeper insights into the management of AA in older patients. Future investigations could focus on several different directions and could include a comparison of adult and pediatric populations with regard to time from onset of symptoms and time to diagnosis and surgery, investigating the clinical presentation of various signs and symptoms in different age groups. Such studies could highlight the needed differences in the awareness of the medical staff, index of suspicion, and priorities. Furthermore, such studies could help develop a more age-adapted scoring system for assessing patients with suspected acute appendicitis. Similarly, future studies could further assess the impact of coexisting comorbidities on the clinical presentation of AA. In addition, future prospective research should collect information regarding surgical technique details; preoperative parameters, such as time from onset of symptoms to surgery; and physiologic and laboratory parameters in order to better understand the existing age-related differences.

### Limitations 

Our study has several limitations. First, the registry cannot identify the parameters of a single patient. Additionally, the database does not include the time from the beginning of symptoms until hospital arrival, time to diagnosis, and time to surgery. Information regarding open versus laparoscopic appendectomy is also lacking in this registry. Rates of SSI have been reported to be affected by the choice of an open vs. laparoscopic approach, and this may have influenced our results as well. Second, the reported final diagnosis is set upon patient discharge and is not updated after receiving pathology results [26]. In addition, the patient population reflects only a cohort of patients within the USA. AA in most other parts of the globe is managed differently and carries different outcomes. Therefore, our results can not be fully implemented for low- and middle-income countries. Lastly, this is a retrospective study using a national registry; and thus, it might have had difficulties in including precise post-discharge data, which may have led to an underestimation of the incidence of SSI. 

This study has also several strengths. To the best of our knowledge, this is the largest study performed on AA patients. This work includes a wide patient age range, from 18 to 90 years, with a large difference in the number of patients between the two study groups. Nevertheless, we believe that the variability between groups and the lack of some relevant clinical information can probably be minimalized, if not nullified, by the large sample size provided by the registry. 

## 5. Conclusions

This comprehensive analysis sheds light on the characteristics and outcomes of AA in older individuals compared to their younger counterparts. Our findings reveal a higher incidence of AA in the older population than previously reported, emphasizing the importance of considering AA as a differential diagnosis in this group of patients. While significant comorbidities were prevalent among older patients, only hypertension, heart failure, and obesity were identified as individual risk factors for surgical site infections in elderly patients, and only heart failure was found to be a risk factor for superficial wound infection in this group. Contrarily, in younger patients, most comorbidities, albeit rare in this group, were found to be significant risk factors for surgical site infections in younger patients. The specific individual risk factors for SSI between age groups highlight the unique features of AA in older patients. These results underscore the specific and somewhat unique characteristics of AA in older patients. Further research is essential to elucidate optimal management strategies and improve outcomes in this vulnerable patient population. Moreover, the octogenarian population truly makes a difference compared to the rest. An octo/nonagenarian population might be a new and different venue, and we believe the studies on this unique population may have a significant contribution to the current literature.

## Figures and Tables

**Table 1:** Demographics, chronic health conditions, and rates of complicated appendicitis.

	Group A (*n* = 390,050)	Group B (*n* = 148,350)	*p*-Value
Male gender (%)	206,800 (53.0%)	72,395 (48.8%)	<0.01
Average age (range)	38 (18–59)	70.6 (60–90)	-
Appendectomy	334,270 (85.7%)	111,705 (75.3%)	<0.01
Complicated AA (%)	388,040 (99.5%)	147,810 (99.6%)	<0.01
Dementia (%)	195 (0.05%)	5460 (3.7%)	<0.01
CVD (%)	995 (0.3%)	3055 (2.1%)	<0.01
CKD (%)	6350 (1.6%)	17,475 (11.8%)	<0.01
HTN (%)	70,840 (18.1%)	96,515 (65.1%)	<0.01
HF (%)	4100 (1.0%)	13,205 (8.9%)	<0.01
Obesity (%)	55,630 (14.3%)	22,900 (15.4%)	<0.01
DM (%)	30,880 (7.9%)	33,280 (22.4)	<0.01

AA—acute appendicitis; CVD—cerebrovascular disease; CKD—chronic kidney disease; HTN—hypertension; HF—heart failure; DM—diabetes mellitus.

**Table 2:** Clinical outcomes in patients who underwent an appendectomy.

		Group A (*n* = 334,270)	Group B (*n* = 111,705)	*p*-Value
Superficial wound infection (%)	Complicated AA	515 (0.15%)	335 (0.3%)	<0.01
	Uncomplicated AA	0	0	-
Systemic infection (%)	Complicated AA	2335 (0.7%)	1020 (0.9%)	<0.01
	Uncomplicated AA	10 (0.5%)	5 (1.3%)	0.163
Mortality (%)	Complicated AA	180 (0.05%)	710 (0.6%)	<0.01
	Uncomplicated AA	0	0	-
Mean LOS (days, range)	Complicated AA	2.77 (0–159)	4.56 (0–97)	<0.01
	Uncomplicated AA	1.8 (0–17)	3.25 (0–28)	<0.01

AA—acute appendicitis; LOS—length of hospital stay.

**Table 3:** Sub-analysis of results in Group B.

		Group B1 (*n* = 77,985)	Group B2 (*n* = 70,365)	*p*-Value
Superficial wound infection (%)	Complicated AA	155 (0.2%)	180 (0.3%)	0.02
	Uncomplicated AA	0	0	-
Systemic infection (%)	Complicated AA	555 (0.9%)	465 (0.9%)	0.821
	Uncomplicated AA	0	5 (2.3%)	0.072
Mortality (%)	Complicated AA	150 (0.2%)	560 (1.1%)	<0.01
	Uncomplicated AA	0	0	-
Mean LOS (days, range)	Complicated AA	4.07 (0–70)	5.15 (0–97)	<0.01
	Uncomplicated AA	2.94 (0–28)	3.49 (0–22)	0.101

AA—acute appendicitis; LOS—length of hospital stay.

**Table 4:** Rates of chronic health conditions in patients with superficial wound infection and systemic infection.

		**Superficial Wound Infection** **(*n* = 515)**	**No Superficial Wound Infection** **(*n* = 333,755)**	***p*-Value**	**Systemic Infection** **(*n* = 2345)**	**No Systemic** **Infection (*n* = 331,925)**	***p*-Value**
Group A	Dementia (%)	0 (0%)	140 (0.04%)	0.642	0 (0%)	140 (0.04%)	0.32
	CVD (%)	10 (1.9%)	695 (0.2%)	<0.01	5 (0.2%)	700 (0.2%)	0.98
	CKD (%)	45 (8.7%)	4720 (1.4%)	<0.01	45 (1.9%)	4720 (1.4%)	0.043
	HTN (%)	205 (39.8%)	58,250 (17.5%)	<0.01	685 (29.2%)	57,770 (17.4%)	<0.01
	HF (%)	35 (6.8%)	2855 (0.9%)	<0.01	70 (3.0%)	2820 (0.8%)	<0.01
	Obesity (%)	150 (29.1%)	47,835 (14.3%)	<0.01	440 (18.8%)	47,545 (14.3%)	<0.01
	DM (%)	95 (18.4%)	24,570 (7.4%)	<0.01	310 (13.2%)	24,355 (7.3%)	<0.01
		**Superficial Wound Infection** **(*n* = 335)**	**No Superficial Wound Infection** **(*n* = 111,370)**	***p*-Value**	**Systemic Infection** **(*n* = 1025)**	**No Systemic** **Infection (*n* = 110,680)**	***p*-Value**
Group B	Dementia (%)	5 (1.5%)	3215 (2.9%)	0.128	35 (3.4%)	3185 (2.9%)	0.306
	CVD (%)	10 (3.0%)	1975 (1.8%)	0.094	15 (1.5%)	1970 (1.8%)	0.445
	CKD (%)	40 (11.9%)	11,950 (10.7%)	0.475	110 (10.7%)	11,880 (10.7%)	0.998
	HTN (%)	230 (68.7%)	71,600 (64.3%)	0.096	735 (71.7%)	71,095 (64.2%)	<0.01
	HF (%)	55 (16.4%)	8545 (7.7%)	<0.01	140 (13.7%)	8460 (7.6%)	<0.01
	Obesity (%)	65 (19.4%)	17,755 (15.9%)	0.084	260 (25.4%)	17,560 (15.9%)	<0.01
	DM (%)	75 (22.4%)	23,770 (21.3%)	0.641	235 (22.9%)	23,610 (21.3%)	0.215

CVD—cerebrovascular disease; CKD—chronic kidney disease; HTN—hypertension; HF—heart failure; DM—diabetes mellitus.

**Table 5:** Multivariate analysis.

		OR	95% CI		*p*-Value
			Lower	Upper	
Group A Superficial wound infection	CVD	4.019	2.101	7.687	<0.01
	CKD	2.336	1.632	3.342	<0.01
	HTN	2.086	1.697	2.563	<0.01
	HF	2.931	1.988	4.324	<0.01
	Obesity	1.783	1.459	2.181	<0.01
	DM	1.261	0.972	1.634	0.08
Group A Systemic infection	HTN	1.689	1.529	1.865	<0.01
	HF	2.224	1.733	2.855	<0.01
	Obesity	1.133	1.017	1.263	0.024
	DM	1.359	1.19	1.552	<0.01
	Complicated AA	0.816	0.438	1.522	0.522
Group B Superficial wound infection	HF	2.364	1.769	3.159	<0.01
Group B Systemic infection	HTN	1.252	1.088	1.439	0.02
	HF	1.699	1.415	2.04	<0.01
	Obesity	1.673	1.449	1.932	<0.01
	Complicated AA	1.389	0.573	3.365	0.467

CVD—cerebrovascular disease; CKD—chronic kidney disease; HTN—hypertension; HF—heart failure; DM—diabetes mellitus; AA—acute appendicitis.

## Data Availability

All data were taken from the National (Nationwide) Inpatient Sample (NIS). This database is part of a family of databases and software tools developed for the Healthcare Cost and Utilization Project (HCUP). The NIS is the largest publicly available all-payer inpatient healthcare database designed to produce U.S. regional and national estimates of inpatient utilization, access, cost, quality, and outcomes. Unweighted, it contains data from around 7 million hospital stays each year. Weighted, it estimates around 35 million hospitalizations nationally. Developed through a federal–state–industry partnership sponsored by the Agency for Healthcare Research and Quality (AHRQ), HCUP data inform decision-making at the national, state, and community levels.
